# Long-term follow-up of adolescent depression. A population-based study

**DOI:** 10.3109/03009730903572057

**Published:** 2010-03-10

**Authors:** Hannes Bohman, Ulf Jonsson, Aivar Päären, Anne-Liis von Knorring, Gunilla Olsson, Lars von Knorring

**Affiliations:** ^1^Department of Neuroscience, Child and Adolescent Psychiatry, Uppsala UniversitySweden; ^2^Department of Neuroscience, Psychiatry, Uppsala UniversitySweden

**Keywords:** Adolescent depression, child-birth, health care, long-term follow-up, population-based sample, pregnancy

## Abstract

Adolescent depression is common. Earlier studies indicate that relapses and recurrences are common. But many questions are still unanswered. The aim of the present study has been to follow subjects with adolescent depressions, identified in a population-based study, over a 15-year period. Subjects with adolescent depression (*n* = 362) and a comparison group (*n* = 250) were followed in the National Swedish registers.

The formerly depressed females had significantly more out-patient visits, and a significantly higher proportion (78.4% versus 69.6%) had at least one out-patient visit. Among the males, no significant differences were found as concerns out-patient visits. The formerly depressed females had significantly more in-patient stays (3.6 versus 2.4) and a significantly higher total number of in-patient days (27.4 versus 10.1). A significantly higher proportion had in-patient days due to mental disorders (9.5% versus 4.6%), in particular anxiety disorders (4.9% versus 1.0%). As concerns the males, a significantly higher proportion had in-patient days due to mental disorders (16.5% versus 1.8%), in particular alcohol and drug abuse (7.6% versus 0%).

Among the formerly depressed females there were no significant differences against the comparison group as concerns the proportion of being a mother, number of children per woman, or age at first child. However, a significantly higher proportion of the formerly depressed females had had different, usually mild, disorders related to pregnancy (8.6% versus 0.6%). The children of the women with adolescent depressions were not affected.

## Introduction

According to the World Health Organization (1), depression is the leading cause of burden of disease in middle- and high-income countries. During recent years, the lifetime rates of major depression have increased, and the age of onset has decreased (2). In adolescence, the 12-month prevalence of major depression ranges from 3% to 17% (2–8). Furthermore, sub-syndromal depressive disorders are common in adolescence (7,9). In a series of studies, it has been demonstrated that subjects with sub-syndromal depressions have significant clinical and functional impairment, and the effects on the quality of life of the subjects are pronounced (9–13).

There are some studies in which clinical (14–16) or epidemiological (17–26) samples have been followed over time. Most studies indicate that the adolescents recover from the index episode but that the rate of relapses and recurrences is high. There are also results indicating disturbances not only in mental health but also in physical health. Keenan-Miller *et al*. (26) reported that early adolescent depression continued to be associated with poorer interviewer-rated health, poorer self-perceived general health, higher health care utilization, and increased work impairment due to physical health. Co-morbid disorders seem to predict a more severe course. Furthermore, it has been demonstrated that the long-term outcome is very similar in subjects with adolescent major depressive disorders and in subjects with sub-syndromal depressive disorders (18,27). Almost no studies have reported the number of children and complications of pregnancy and child-birth of individuals with adolescent depression, but there are reports indicating that among patients with teenage onset of depression fewer were later married (28).

However, the course may be episodic or chronic (29), and there are also subjects with single episodes of depression in adolescence who do not have relapses or recurrences (30–32). Many important questions are still unanswered concerning the prognosis of adolescent depression.

In the present study, the aim has been to follow subjects with adolescent depressions, identified in an epidemiological study, over a 15-year period as concerns morbidity.

## Material and methods

### Subjects

In 1990 to 1991, the total population of 16–17-year-olds in a Swedish university city was screened for depression. There were 2,465 adolescents in the age group, and 2,300 (93%) participated in a screening with two self-evaluations of depression, the Beck Depression Inventory (BDI) and the Centre for Epidemiological Studies–Depression Scale for Children (CES-DC). Subjects with high scores (BDI ≥ 16 or CES-DC ≥ 30 + BDI ≥ 11), or who reported a suicide attempt, were diagnostically interviewed with the Diagnostic Interview for Children and Adolescents in the revised form according to DSM-III-R for adolescents (DICA-R-A). For each subject with high scores, a same-sex classmate with low scores on the screening questionnaires and without attempted suicide was selected and interviewed in the same manner, in order to create a comparison group.

In all, 631 adolescents were interviewed. However, 16 of the subjects refused to participate in any follow-up study. Thus, 615 subjects were willing to participate in a follow-up. Fifty-five out of the 307 subjects in the comparison group were found to suffer from a depressive disorder at the interview. Thus, a group of 363 subjects was formed from subjects with high scores on the screening instruments or who had attempted suicide or with a depressive disorder at the interview. A total of 252 subjects remained in the comparison group. One subject in the group of depressed adolescents and two subjects in the comparison group had incomplete personal identification numbers. Thus, in total, it was possible to follow up 612 subjects, 261 with adolescent depression and 101 with sub-syndromal depression or dysthymia. As an earlier study (18) clearly has demonstrated that the long-term outcome is about the same in adolescent major depression and in adolescent sub-syndromal depression, and as a pilot study in the present series revealed the same results, the group with adolescent depressions was kept as one entity. Details about the screening procedure and about how the groups were formed can be seen in Table I.

**Table I.** Participants in the original epidemiological study 1991–1992 (7) and selected for a long-term follow up 15 years later.

Total number of 16-year-olds in the city of Uppsala: *n* = 2,465		
Participated in the screening procedure: *n* = 2,300		
Delivered complete screening questionnaires: *n* = 2,270		
	Selected due to high scores on the Beck Depression Inventory (BDI) or Center for Epidemiological Studies scale (CES) or had attempted suicide	Selected as controls
Selected for interview	*n* = 355	*n* = 355
Interviewed (structured interview by means of DICA)	*n* = 314	*n* = 317
Refused to participate in follow up	*n* = 6	*n* = 10
Interviewed and willing to participate in follow-up	*n* = 308	*n* = 307
Depressed at DICA interview		*n* = 55
	Depressed at screening or at interview or having at least one suicide attempt: *n* = 363	Controls (not depressed at screening or interview and without any suicide attempt): *n* = 252
Incomplete personal identification nr	*n* = 1	*n* = 2
Included in follow-up	*n* = 362	*n* = 250
Men/Women	80/282	56/194
Age at follow up, years	31.6 ± 0.7	31.6 ± 0.6
Major depression at first investigation Females/Males	213/48	
Dysthymia and sub-syndromal depression at first investigation Females/Males	70/31	

DICA: Diagnostic Interview for Children and Adolescents in the revised form according to DSM-III-R for adolescents (DICA-R-A).

### The registers

The Swedish National Health and Welfare Board keeps the official registers concerning health and sickness in Sweden. Some of these registers were used in the present study.

#### The national patient register

Since 1987, the register includes information concerning episodes of in-patient care in Sweden. Out-patient visits in surgery are available since 1997, and other out-patient visits to specialized doctors are registered since 2001. Thus, the register data do cover only a part of the 15-year follow-up period.

The total number of drop-outs for somatic short-time care for the period 1987–1991 has been estimated to be less than 2%.

For all records reported to the national patient register a data control is run. A check is made that compulsory variables are reported, e.g. personal identification number, hospital, and main diagnosis. A check is also made that codes for different variables and dates have valid values. Some obviously incorrect data is corrected in connection with the quality controls. The personal identification number (PIN) makes it possible to follow a patient between different hospitals and over time. The number of stays in 2003 with missing personal identification number was 0.7%.

#### The mother–child register and the medical birth register

By means of these registers, all children belonging to a separate mother can be identified, and information can be collected concerning medical complications during pregnancy and delivery. Furthermore, there is information concerning the health of the new-born children.

### Statistics

Differences between means have been tested by means of the Mann-Whitney test. Differences between frequency distributions were tested by means of the chi-square test. When less than five subjects were expected in one cell, the Fisher exact test was used.

### Ethics

To keep the integrity of the subjects and to avoid any drop-outs, all information was collected group-wise. Thus, data were available as concerning health care consumption for the formerly depressed patients and the comparison group but not as concerns individual subjects. The study was approved by the Ethical Vetting Board in Uppsala.

## Results

As concerns the formerly depressed females, they had significantly more out-patient visits, and a significantly higher proportion, 78.4% versus 69.6%, had at least one registered out-patient visit (Table II). A significantly higher proportion of the formerly depressed females had certain infectious and parasitic diseases (4.6% versus 1.0%), diseases of the nervous system (6.4% versus 2.1%), diseases of the digestive system (9.2% versus 4.1%), injuries, poisonings, and certain other consequences of external causes including attempted suicide and accidents (16.3% versus 8.2%) (Table II). As concerns the males, no significant differences were found between formerly depressed males and the comparison group as concerns out-patient visits (Table II).

**Table II.** Number of out-patient visits, proportion with at least one out-patient visit, as well as the proportion of subjects with at least one out-patient visit due to a specific diagnostic subgroup.

	Females			Males		
	Adolescent depressions *n* = 283	Controls *n* = 194	Significance	Adolescent depressions *n* = 79	Controls *n* = 56	Significance
Number of out-patient visits	7.5 ± 12.0	4.1 ± 8.3	*z* = 4.27; *P* <0.0001	3.6 ± 5.4	3.4 ± 6.7	n.s.
Proportion with at least one out-patient visit	222 (78.4%)	135 (69.6%)	χ^2^ = 4.80; *P* <0.05	52 (65.8%)	34 (60.7%)	n.s.
Certain infectious and parasitic diseases	13 (4.6%)	2 (1.0%)	χ^2^ = 4.80; *P* <0.05	4 (5.1%)	1 (1.8%)	n.s.
Neoplasms	5 (1.8%)	-	n.s.	-	1 (1.8%)	n.s.
Diseases of the blood and blood-forming organs and certain disorders involving the immune mechanism	17 (6.0%)	14 (7.2%)	n.s.	3 (3.8%)	3 (5.4%)	n.s.
Endocrine, nutritional, and metabolic diseases	12 (4.2%)	5 (2.6%)	n.s.	1 (1.3%)	-	n.s.
Organic, including symptomatic, mental disorders	32 (11.3%)	12 (6.2%)	n.s.	9 (11.4%)	2 (3.6%)	n.s.
Mental and behavioral disorders due to psychoactive substance use	4 (1.4%)	0 (0.0%)	n.s.	5 (6.3%)	0 (0.0%)	n.s.
Schizophrenia, schizotypal and delusional disorders	-	-	-	-	1 (1.8%)	n.s.
Mood (affective) disorders	12 (4.2%)	6 (3.1%)	n.s.	2 (2.5%)	1 (1.8%)	n.s.
Neurotic, stress-related and somatoform disorders	14 (4.9%)	8 (4.1%)	n.s.	2 (2.5%)	-	n.s.
Behavioral syndromes associated with physiological disturbances and physical factors	6 (2.1%)	2 (1.0%)	n.s.	-	-	-
Disorders of adult personality and behavior	3 (1.1%)	1 (0.5%)	n.s.	1 (1.3%)	-	n.s.
Mental retardation	-	-	-	1 (1.3%)	-	n.s.
Behavioral and emotional disorders with onset usually occurring in childhood and adolescence	1 (0.4%)	-	n.s.	-	-	-
Mental and behavioral disorders	32 (11.3%)	12 (6.2%)	n.s.	9 (11.4%)	2 (3.6%)	n.s.
Diseases of the nervous system	18 (6.4%)	4 (2.1%)	χ^2^ = 4.83; *P* <0.05	3 (3.8%)	1 (1.8%)	n.s.
Diseases of the eye and adnexa	10 (3.5%)	2 (1.0%)	n.s.	1 (1.3%)	1 (1.8%)	n.s.
Diseases of the ear and mastoid process	9 (3.2%)	1 (0.5%)	n.s.	4 (5.1%)	1 (1.8%)	n.s.
Diseases of the circulatory system	6 (2.1%)	5 (2.6%)	n.s.	2 (2.5%)	0 (0.0%)	n.s.
Diseases of the respiratory system	20 (7.1%)	13 (6.7%)	n.s.	3 (3.8%)	1 (1.8%)	n.s.
Diseases of the digestive system	26 (9.2%)	8 (4.1%)	χ^2^ = 4.46; *P* <0.05	7 (8.9%)	6 (10.7%)	n.s.
Diseases of the skin and subcutaneous tissue	30 (10.6%)	15 (7.7%)	n.s.	5 (6.3%)	4 (7.1%)	n.s.
Diseases of the musculoskeletal system and connective tissue	31 (11.0%)	15 (7.7%)	n.s.	9 (11.4%)	6 (10.7%)	n.s.
Diseases of the genitourinary system	73 (25.8%)	37 (19.1%)	n.s.	11 (13.9%)	5 (8.9%)	n.s.
Pregnancy, child-birth, and the puerperium	71 (25.1%)	40 (20.6%)	n.s.	-	-	-
Symptoms, signs, and abnormal clinical and laboratory findings	70 (24.7%)	37 (19.1%)	n.s.	11 (13.9%)	4 (7.1%)	n.s.
Injury, poisoning, and certain other consequences of external cause	46 (16.3%)	16 (8.2%)	χ^2^ = 6.53; *P* <0.02	29 (36.7%)	17 (30.4%)	n.s.
Others or unspecified	133 (47.0%)	76 (39.2%)	n.s.	19 (24.1%)	17 (30.4%)	n.s.

χ^2^ = chi-square

Among the females, the formerly depressed females had significantly more in-patient stays (3.6 versus 2.4) and a significantly higher total number of in-patient days (27.4 versus 10.1). A significantly higher proportion of the formerly depressed females had in-patient days due to mental and behavioral disorders (9.5% versus 4.6%), in particular anxiety disorders (4.9% versus 1.0%). As concerns the formerly depressed males, a significantly higher proportion had in-patient days due to mental and behavioral disorders (16.5% versus 1.8%), in particular alcohol and drug abuse (7.6% versus 0%) (Table III).

**Table III.** Number of in-patient stays, total number of in-patient days and frequency of subjects hospitalized due to diseases in different diagnostic groups.

	Females			Males		
	Adolescent depressions *n* = 283	Controls *n* = 194	Significance	Adolescent depressions *n* = 79	Controls *n* = 56	Significance
Number of subjects ever hospitalized	232 (82.0%)	147 (75.8%)	n.s.	54 (68.4%)	39 (69.6%)	n.s.
Number of times as in-patient	3.6 ± 5.1	2.4 ± 2.4	*z* = 2.15; *P* <0.05	2.4 ± 3.1	2.2 ± 3.3	n.s.
Total number of in-patient days	27.4 ± 141.6	10.1 ± 22.5	*z* = 1.96; *P* <0.05	13.9 ± 48.6	11.6 ± 36.0	n.s.
Infectious diseases	27 (9.5%)	14 (7.2%)	n.s.	9 (11.4%)	9 (16.1%)	n.s.
Neoplasms	4 (1.4%)	3 (1.5%)	n.s.	-	2 (3.6%)	n.s.
Diseases of the blood and the immune system	7 (2.5%)	2 (1.0%)	n.s.	1 (1.3%)	-	n.s.
Endocrine and metabolic diseases	4 (1.4%)	5 (2.6%)	n.s.	-	1 (1.8%)	n.s.
Mental and behavioral disorders	27 (9.5%)	9 (4.6%)	χ^2^ = 3.96; *P* <0.05	13 (16.5%)	1 (1.8%)	χ^2^ = 7.59; *P* <0.01
Depressive disorders	8 (2.8%)	4 (2.1%)	n.s.	4 (5.1%)	-	n.s.
Anxiety disorders	14 (4.9%)	2 (1.0%)	χ^2^ = 5.45; *P* <0.05	4 (5.1%)	-	n.s.
Alcohol and drug abuse	5 (1.8%)	2 (1.0%)	n.s.	6 (7.6%)	-	*P* = 0.04
Suicidal behavior	13 (4.6%)	4 (2.1%)	n.s.	5 (6.3%)	3 (5.4%)	n.s.
Diseases of the nervous system	8 (2.8%)	2 (1.0%)	n.s.	0	2 (3.0%)	n.s.
Diseases of the eye	7 (2.5%)	4 (2.1%)	n.s.	1 (1.3%)	-	n.s.
Diseases of the ear	4 (1.4%)	1 (0.5%)	n.s.	3 (3.8%)	-	n.s.
Diseases of the circulatory system	2 (0.7%)	4 (2.1%)	n.s.	4 (5.1%)	1 (1.8%)	n.s.
Diseases of the respiratory system	56 (19.8%)	31 (16.0%)	n.s.	17 (21.5%)	11 (19.6%)	n.s.
Diseases of the digestive system	39 (13.8%)	21 (10.8%)	n.s.	12 (15.2%)	7 (12.5%)	n.s.
Diseases of the skin	9 (3.2%)	4 (2.1%)	n.s.	1 (1.3%)	0	n.s.
Diseases of the musculoskeletal system	15 (5.3%)	7 (3.6%)	n.s.	4 (5.1%)	4 (7.1%)	n.s.
Diseases of the genitourinary system	18 (6.4%)	17 (8.8%)	n.s.	5 (6.3%)	0	n.s.
Symptoms, signs	56 (19.8%)	27 (13.9%)	n.s.	14 (17.7%)	11 (19.6%)	n.s.
Injuries and poisoning	50 (20.8%)	35 (18.0%)	n.s.	20 (25.3%)	14 (25.0%)	n.s.
External causes of morbidity	68 (24.0%)	37 (19.1%)	n.s.	22 (27.8%)	16 (28.6%)	n.s.

χ^2^ = chi-square.

Among the formerly depressed females there were no significant differences compared to the comparison group as concerns having any children, number of children per woman, or age at first child (Table IV A). However, a significantly higher proportion of the formerly depressed females had had maternal disorders predominantly related to pregnancy (8.6% versus 0.6%), complications predominantly related to the puerperal period (5.3% versus 1.2%), and any complication whatsoever related to pregnancy or delivery (57.4% versus 45.2%) (Table IV B).

**Table IV.** Number of childen as well as diseases of the mother and child during pregnancies and deliveries in women with former adolescent depression and controls.

A. Number of deliveries and number of children.			
	Women with adolescent depression *n* = 283	Controls *n* = 194	Significance
Giving birth to at least one child	133 (47.0%)	90 (46.4%)	n.s.
Number of children per woman	0.81 ± 1.00	0.82 ± 0.98	n.s.
Age at first child's birth (years)	25.6 ± 3.5	26.1 ± 3.0	n.s.

B. Diseases of the mothers.			

	Women with adolescent depression *n* = 245 pregnancies	Controls *n* = 166 pregnancies	Significance

Edema, proteinuria and hypertensive disorders in pregnancy, during child-birth and the puerperium	11 (4.5%)	7 (4.2%)	n.s.
Other maternal disorders predominantly related to pregnancy	21 (8.6%)	1 (0.6%)	χ^2^ = 12.40; *P* <0.001
Maternal care related to the fetus and amniotic cavity and possible delivery problems	41 (16.8%)	25 (15.1%)	n.s.
Complications of labor and delivery	92 (37.4%)	53 (31.9%)	n.s.
Complications predominantly related to the puerperal period	13 (5.3%)	2 (1.2%)	χ^2^ = 4.70; *P* <0.05
Other obstetric conditions, not elsewhere classified	19 (7.7%)	7 (4.2%)	n.s.
Any complication	140 (57.4%)	75 (45.2%)	χ^2^ = 5.89; *P* <0.02

χ^2^ = chi-square.

The children of the women with adolescent depressions did not have any increased risk of complications related to maternal factors or any complications of pregnancy, labor, or delivery (detailed data not shown).

### Major depressive disorder versus sub-syndromal depression

In the areas where significant differences were found between formerly depressed subjects and subjects in the comparison group, a further test was done to investigate possible differences between subjects with former major depressive episodes and subjects with former sub-syndromal depressions or dysthymia. The females with former major depressive episodes did not differ significantly from females with former sub-syndromal depressions or dysthymia as concerns number of out-patient visits (7.4 ± 11.0 versus 7.4 ± 14.9, n.s.), proportion with at least one out-patient visit (78.9% versus 77.1%, n.s.), certain infectious and parasitic diseases (3.8% versus 7.1%, n.s.), diseases of the nervous system (7.5% versus 2.9%, n.s.), diseases of the digestive system (8.9% versus 11.4%, n.s.), or injuries and poisoning (17.8% versus 11.4%). As concerns in-patient stays, the females with former major depressive episodes did not differ significantly from females with former sub-syndromal depressions or dysthymia regarding number of times as in-patients (3.8 ± 5.7 versus 2.7 ± 3.0, n.s.) or number of in-patient days (31.4 ± 158.7 versus 15.3 ± 65.7, n.s.). The pregnant women with former major depressive episodes did not differ significantly from pregnant women with former sub-syndromal depressions or dysthymia as concerns maternal disorders predominantly related to pregnancy (9.1% versus 6.9%, n.s.), complications predominantly related to puerperium (5.3% versus 5.2%, n.s.), or any complication during pregnancy (58.7% versus 53.4%, n.s.).

The males with former major depressive episodes did not differ significantly from males with former sub-syndromal depressions or dysthymia as concerns frequency of mental or behavioral disorders (14.6% versus 19.4%, n.s.) or alcohol and drug abuse (6.2% versus 9.7%, n.s.).

However, the females with former major depressive episodes did differ significantly from females with former sub-syndromal depressions or dysthymia as concerns frequency of mental and behavioral disorders (12.7% versus 0%, *P* <0.001) and frequency of anxiety disorders (6.6% versus 0%, *P* = 0.017).

## Discussion

The main findings of the present study are that females with depressive disorders during adolescence have a higher health care consumption during the 15-year follow-up period. They have more out-patient visits to somatic specialists as compared to women without adolescent depression. A higher proportion has also been hospitalized due to mental and behavioral disorders, in particular anxiety disorders. Thus, the results are in line with earlier follow-up studies from clinical (14–16) and epidemiological (17–26) patient samples indicating relapses and recurrences in anxiety disorders (33) and depressive disorders (31,33), as well as worse emotional and physical health. But it must also be kept in mind that the actual percentages found are an under-estimation of the health care used by the subjects. The national registers used do cover in-patient stays and specialized out-patient care. However, visits to general practitioners are not included. As about 70% of all patients with anxiety or depressive disorders are taken care of by general practitioners (27), the data presented are much too low. However, the comparison between the formerly depressed patients and the subjects in the comparison group without adolescent depression is still of considerable interest as the data quality is equal in both groups.

The subjects with adolescent major depressive disorders did have very similar long-term outcomes to those of the subjects with adolescent sub-syndromal disorders or dysthymia as concerns general ill health and somatic co-morbidity, in line with the results from earlier studies (18,34). However, as concerns a long-term course with more mental and behavioral disorders, in particular anxiety disorders, it was more common in the females with adolescent major depressive disorders.

The increased frequency of certain infectious and parasitic diseases in formerly depressed females already at the age of 30 is of interest as it has been demonstrated that depressed patients in long-term follow-up have an about ten times increased risk of death from infectious disorders (35).

However, in the earlier published follow-up studies, there has been a tendency indicating about the same course in males and females with adolescent depression (36). In the present study, it is a clear result that males with former depressive episodes in adolescence do not have more out-patient or in-patient care due to anxiety disorders or depressive disorders. Instead, they have considerably more in-patient stays due to alcohol and drug abuse. In an extensive prospective and retrospective analysis of the material of the Gotland study 12 years ago it was demonstrated that male depression was frequently masked by irritation, anger, hostile-aggressive-abusive behavior, and alexithymia. Depressive males generally do not seek medical help, instead 20% of the male suicide victims were known to the social welfare system as being drug and alcohol abusers, compared to only 3% of the female suicides (37). We also know that the male adolescents included in the present study had more behavioral disturbances and drug abuse (38). Anxiety disorder was co-morbid to depression in one half and conduct disorder in one quarter of the depressed adolescents. Alcohol was abused by 6.5% and used regularly by another 12%. Other drugs were used by 6.5% of depressed adolescents and not at all by the controls (38). Thus, the males might be taken care of outside the health care system, e.g. in prisons or in treatment homes for alcohol and drug abusers.

The hypothesis concerning a depressive spectrum disorder, including families with depressive disorders in the females and alcoholism in the males, has earlier been presented as concerns depressive disorders with adult onset (39). However, it has not earlier been demonstrated that depressive disorders with adolescent onset tend to follow the same course (36).

It is of considerable interest that females with adolescent depression have the same age as the females in the comparison group when they deliver their first child. Furthermore, as many of the formerly depressed females have at least one child as do the women in the comparison group. However, they do have more complications during pregnancy. Mild complications during pregnancy have earlier been demonstrated in women with anxiety and depression (40) in line with the results of the present study. However, serious pregnancy complications are not increased. Furthermore, there is no increased risk that the fetus or new-born is affected by maternal factors or by complications of pregnancy. Similar results have been reported in women with anxiety and depression (41).

Apart from the differences found between the formerly depressed patients and the comparison group, it is also of interest that there is no increased risk of diseases of the circulatory system. In an earlier study (42), covering a subpopulation of the women included in the present study, we were able to demonstrate thicker carotid intima layer, thinner media layer, and higher intima/media ratio in formerly depressed females. However, according to the present results they do not seem to have clinical symptoms or signs of circulatory diseases, at least not of a severity to result in out-patient or in-patient care.

Thus, in conclusion, females with adolescent depression, followed for 15 years, do have more mental health problems, in particular anxiety disorders. They do also have somewhat more complications during pregnancy and delivery, but they have the same number of children, and as new-borns they are not affected. On the other hand, the males with adolescent depressions seem to end up with more alcohol and drug abuse.
